# Analysis of the relationship of refractory gout between potential biomarkers and diet structure and lifestyle based on ^1^H-NMR

**DOI:** 10.1186/s13018-024-04540-2

**Published:** 2024-01-19

**Authors:** Liang Weidong, Chen Liuting, Cheng Xiangcong, Peng Jianhong, Ye Xueying

**Affiliations:** 1 Department of Rheumatology, Dongguan Hospital of Traditional Chinese Medicine, Dongguan, 523200 China; 2 Dongguan Hospital of Traditional Chinese Medicine, Guangzhou University of Chinese Medicine, Guangzhou, 510006 China

**Keywords:** Refractory gout, Metabolomics, Diet structure, Lifestyle

## Abstract

**Objective:**

We investigated the different life styles among the diet structures and exercise patterns of 100 patients with refractory gout and 79 healthy volunteers; of these, we selected 39 patients and 20 healthy volunteers for serum proton magnetic resonance (^1^H-NMR) metabolic network detection. We determined the potential biomarkers of refractory gout and attempted to explore the relation between potential biomarkers and diet structures and exercise patterns.

**Methods:**

The study employed a questionnaire survey to analyze diet structures and exercise patterns from 100 patients of refractory gout and 79 healthy volunteers. At the same time, using ^1^H-NMR metabolic technology to analyze the metabolites present in the serum samples obtained from 39 patients of refractory gout (group B) and 20 healthy subjects (group A). Employing MestReNova (Version 8.0.1) to analyze the metabolites maps, collecting the NMR results, further importing into SIMCA-P+ 14.0 software (Umetrics, Sweden) for principal component analysis (PCA), partial least squares discriminant analysis (PLS-DA), and orthogonal partial least squares discriminant analysis (OPLS-DA) statistical analysis. Combining patterns recognition and multivariate statistics, potential biomarkers were searched. Other experimental data, including creatinine and adiponectin, were counted by the SPSS21.0. The measurement data were expressed by X ± S and *t* test. The counting data were expressed in percent and performed by *X*^2^ test.

**Results:**

Our results revealed that patients with gout tended to be obese, and there were differences in their lifestyle with exercise, sleep, and smoking, as well as in their preference for fructose drinks, alcohol, and total and structural distribution of meat, milk, eggs, and so on when compared with the healthy volunteers. Importantly, we found the adiponectin in the gout group was lower as compared to the healthy group. Further, metabolomics in combination with KEGG analysis revealed that the biosynthesis of aminoacyl tRNA, biosynthesis of valine, leucine, and isoleucine, metabolism of alanine, aspartic, and glutamate, metabolism of glycine, serine, and threonine, phenylalanine, glycolysis/gluconeogenesis, ketone body synthesis and degradation, metabolism of d‐glutamine, citric acid cycle (TCA cycle), triglyceride metabolism, and others could be used as specific biomarkers of this disease.

**Conclusion:**

Recurrent refractory gout and formation of tophus may be related to the diet structures and lifestyles between the patients and the healthy people, and their abnormal metabolic network may be related to the disorder of mitochondrial energy metabolism, which further results in abnormal metabolism of glucose, lipids, amino acids, and deposition of uric acid in joints, peripheral connective tissue, and kidney, inducing an inflammatory response.

## Introduction

Gout is one of the most common crystal‐related arthropathies, which involves the deposition of monosodium urate (MSU) crystals [1]. Pathologically, it involves the deposition of uric acid, which often leads to a series of injuries in the tissues and organs, such as the joint cavity, blood vessels, heart, and kidney. It has been previously reported that gout was often associated with hyperlipidemia, hypertension, diabetes, arteriosclerosis, and coronary heart disease [2, 3]. Refractory gout refers to recurrent attacks of gout arthritis, wherein conventional uric acid drugs fail to meet the required target. Chronic multiple arthritis usually involves the formation of tophus, further the potential occurrences of uric acid kidney stones and joint degeneration should also be considered [4]. According to 2020 global gout epidemiology data, there has been an increase in the incidence of gout, and its prevalence rate has increased significantly in the past few years [1]. Prevalence of hyperuricemia was recorded to be 2.6–36.0% in different ethnic groups, wherein the prevalence of gout was recorded to be 0.03–15.30% [5]. Importantly, there is an increase in the prevalence of both hyperuricemia and gout in recent years [6]. Effective regulation of lifestyle and diet structure is beneficial to control and prevent gout attacks. Metabolomics generally refers to the quantitative analysis of all the metabolites present in an organism. It evaluates changes in metabolites during different states of the organism and aids in revealing the metabolic essence of an organism's life activities [7]. The present study surveyed 100 refractory gout patients’ and 79 healthy volunteers’ diet structures and lifestyle, and further selected the top 39 patients with average frequency of attacks in recent 3 years from 100 patients and 20 cases of healthy volunteers to study their serum metabolic network, determined refractory gout potential biomarkers, and explored the relation between potential biomarkers and diet structures and exercise patterns to guide patients adjust their diet structures and exercise patterns.

## Cases and reagents

### Case collection

We adopted a convenient sampling method to conduct a questionnaire survey by offline and online questionnaires. A total of 110 questionnaires were distributed to refractory gout inpatients and outpatients visiting the Dongguan Hospital of Traditional Chinese Medicine between January and November 2021, of which 100 questionnaires were effectively recovered, with an effective recovery rate of 90.9%. Furthermore, we selected the top 39 patients with the highest frequency of gout attacks in the past 3 years (31 men, 80%; 8 women, 20%; average age: 50.17 ± 12.73 years) as Group B for the metabolic network analysis.

A total of 90 questionnaires were distributed to health volunteers from the physical examination center of the Dongguan Hospital of Traditional Chinese Medicine between January and November 2021, of which 79 questionnaires were effectively recovered, with an effective recovery rate of 87.8%. Furthermore, we randomly selected 20 patients (16 men, 80%; 4 women, 20%; average age: 48.47 ± 12.51 years) as Group A according to the gender and age of Group B. This study was reviewed by the Ethics Committee of the Dongguan Hospital of Traditional Chinese Medicine, and all participants are required to sign their informed consent.

### Subject inclusion criteria

#### Refractory gout diagnostic criteria

The diagnostic criteria for refractory gout according to the “2015 Gout New Classification Criteria from ACR/EULAR”: (1) The serum uric acid level was ≥ 360umol/L despite sufficient dosage and course treatment of conventional urate-lowering drugs alone or in combination; (2) occurrence of recurrent gout attack more than 2 times/years despite standardized treatment; and (3) presentation of persistent and/ or extensive tophus.

#### Patient ranged in age

The age ranged from 20 to 80 years.

### Patient exclusion criteria

Pregnancy or breastfeeding women and psychiatric patients; patients with severe malnutrition or serious damage to the heart, brain, kidney, and hematological system; merging of other rheumatism patients; and patients who did not cooperate with the study process.

### Reagent

Anhydrous sodium hydrogen phosphate (NaH_2_PO_4_) was sourced from the Guangdong Guangxian Reagent Technology Co., Ltd. Sodium phosphate dibasic dihydrate (Na_2_HPO_4_) was obtained from Yueqiao Co., Ltd. Heavy water was sourced from the Hangpu Experimental Equipment Co., Ltd.

### Experimental apparatus

NMR spectrometer with a superconducting magnet (Varian NMR System 500 MHz; American Varian Co., Ltd.); fully automatic biochemical analyzer BS-220 (Mindray); microporous plate oscillator (Wuxi Jieruian Instrument Equipment Co., LTD); NMR TUBE (Ning Hangpu Experimental Equipment Co., LTD); TGL-20 M Desktop high-speed freezing centrifuge (Shanghai Luxiangyi Centrifuge Instrument Co., LTD) were used in this study.

## Experiments and methods

### Sample pretreatment

For serum collection, 6 mL of the blood samples were, respectively, collected from patients and healthy people, at 4 °C and the frequency of 10,000 Hz centrifugal for 15 min. Further, 300 μL of the upper serum was absorbed into the NMR tube, to which added 200 μL of 0.2 mol/L phosphate equilibrium solution (pH 7.4) and 50 μL of heavy water and mixed by shaking.

### NMR experiment

At 25 °C, the pulse sequence was set as Carr-Purcell-Mei boom-Gill, the spectral width as 10,000 Hz, the echo time as 1 ms, 64-K sampling points, 64 cycles, and 128 stacking times of NMR scanning. The NMR signal was transformed into a map by Fourier-transformed spectroscopy.

### Map processing

MestReNova (Version 8.0.1) was used to analyze the map. The lactic acid peak (*δ* 1.33) served as the chemical shift reference peak. Phase and baseline adjustments were made to the obtained map and defined at *δ* 0.5–9.0, with 0.005 PPM as the integral interval region. The regional water peak of *δ* 4.7–5.1 (the integral value was set as zero) was eliminated, normalized to obtain the integral data, and saved in Excel form for the follow multivariate statistical analysis.

### Data processing

The abovementioned integral data were imported into the SIMCA-P + 14.0 software (Umetrics, Sweden) for PCA, PLS-DA, and OPLS-DA statistical analysis. R2 and Q2, as the validation parameters of the evaluation model, were > 50%, indicating that the model was statistically valid. The OPLS-DA results were expressed in the form of a score plot and a loading plot. By analyzed the chemical shift in the integral difference between the groups in the scoring diagram, found the difference contribution value in the load diagram, and the corresponding metabolites in combination with the peak change of the one-dimensional NMR spectrum. Other experimental data were counted by using the SPSS21.0. The measurement data were expressed by *X* ± *S* and *t* test. The counting data were expressed in percent and performed by *X*^2^ test.

## Experimental results

### Survey results

#### General condition between healthy and gout groups

We noted no statistical differences in age and gender between the two groups. The gout patients had higher body mass index (BMI) than the healthy people, and some gout patients were complicated with hypertension and diabetes, showing statistical differences (Table 1).

**Table 1:** General condition between healthy and gout groups

	Healthy group (Group A) (*N* = 79)	Gout group (Group B) (*N* = 100)	*χ*^2^/*t* value	*P* value
Age (year)	47.08 ± 12.91	49.31 ± 13.25	1.133	0.259
Gender [person(%)]			1.356	0.244
Male	53 (67.09%)	75 (75.00%)		
Female	26 (32.91%)	25 (25.00%)		
BMI (kg/m^2^)	21.90 ± 2.23	25.62 ± 3.58	8.081	0.000
Combined with hypertension and diabetes [person (%)]			19.815	0.000
Both	0 (0.00%)	6 (6.00%)		
Hypertension	0(0.00%)	10 (10.00%)		
Diabetes	0 (0.00%)	6 (6.00%)		
Neither	79 (100.00%)	78 (78.00%)		

#### Lifestyle comparison between healthy and gout groups

We recorded significant differences between the two groups in terms of lifestyle, such as exercise patterns, sleep, and smoking (*P* < 0.05) (Table 2).

**Table 2:** Lifestyle comparison between healthy and gout groups

Questions and options	Healthy group (Group A) (*N* = 79)	Gout group (Group B) (*N* = 100)	*χ*^2^ value	*P* value
Q1: How many times you exercise per week? (more than 30 min each time)			1.346	0.718
A. 0 times	26 (32.9%)	31 (31%)		
B. 1 time	20 (25.3%)	30 (30%)		
C. 2 times	18 (22.1%)	17 (17%)		
D. 3 or more times	15 (19.0%)	22 (22%)		
Q2: Which of the following sports do you usually choose? (multiple choice)			42.161	0.000
A. Walking	32 (57.0%)	64 (64%)		
B. Swimming	31 (6.3%)	7 (7%)		
C. Low intensity aerobics	28 (19.0%)	11 (10%)		
D. High intensity aerobic exercise	4 (7.6%)	18 (14%)		
E. High intensity strength training	9 (10.1%)	11 (12%)		
Q3: How many times do you sedentary per week? (more than 6 h per day)			15.197	0.002
A. 0 times	19 (21.4%)	14 (14%)		
B. 1 time	25 (31.6%)	17 (17%)		
C. 2 times	17 (21.5%)	19 (19%)		
D. 3 or more times	18 (22.8%)	50 (50%)		
Q4: How many times do you sleep well per week? (Sleep at least 6 h at night)			6.460	0.091
A. 0 times	5 (6.3%)	6 (6%)		
B. 1 time	5 (6.3%)	17 (17%)		
C. 2 times	14 (17.7%)	23 (23%)		
D. 3 or more times	55 (69.6%)	54 (54%)		
Q5: How many times do you overeat per week? (Subjective judgment)			20.875	0.000
A. 0 times	29 (36.7%)	21 (21%)		
B. 1 time	33 (41.8%)	24 (24%)		
C. 2 times	10 (12.7%)	37 (37%)		
D. 3 or more times	7 (8.9%)	18 (18%)		
Q6: How many milliliters do you drink daily(Including tea, coffee, etc., except beverages) ?			1.159	0.560
A. ≤ 1500	22 (27.8%)	25 (25%)		
B. 1500–2500	46 (58.2%)	55 (55%)		
C. ≥ 2500	11 (13.9%)	20 (20%)		
Q7: Do you smoke?			10.424	0.001
A. Yes	8 (10.1%)	30 (30%)		
B. No	71 (89.9%)	70 (70%)		

#### Diet structures comparison between healthy and gout groups

We noted several differences between gout and healthy groups in terms of the diet structures, such as fructose drink, alcohol, total and structure distribution of meat, milk, and eggs, and there were statistical differences (*P* < 0.05) (Table 3).

**Table 3:** Diet structure comparison between healthy and gout groups

Questions and options	Healthy group (Group A) (*N* = 79)	Gout group (Group B) (*N* = 100)	*χ*^2^ Value	*P* value
Q8: How much alcohol do you drink daily?			11.085	0.011
A. 0 mL	64 (81.05)	60 (60%)		
B. 0−50 mL	12 (15.2%)	23 (23%)		
C. 50−200 mL	2 (2.5%)	12 (12%)		
D. ≥ 200 mL	1 (1.3%)	5 (5%)		
Q9. How many cups of juice and beverages you drink per week?			0.041	0.840
A. ≤ 1cup	47 (59.5%)	58 (58%)		
B. 2–3 cups	32 (40.5%)	42 (42%)		
Q10.How many grain you intake daily?			14.271	0.003
A. ≤ 200 g	25 (31.6%)	15 (15%)		
B. 201−300 g	33 (41.8%)	55 (55%)		
C. 301−400 g	6 (7.6%)	20 (20%)		
D. ≥ 401 g	15 (19.0%)	10 (10%)		
Q11. How many potatoes you intake daily?			3.750	0.153
A. ≤ 30 g	44 (55.7%)	68 (68%)		
B. 31−60 g	25 (31.6%)	26 (26%)		
C. ≥ 61 g	10 (12.7%)	6 (6%)		
Q12. How many meat you intake daily?			21.854	0.000
A. ≤ 60 g	32 (40.5%)	16 (16%)		
B. 61−120 g	42 (53.2%)	55 (55%)		
C. ≥ 121 g	5 (6.3%)	29 (29%)		
Q13. What kind of meat you usually eat? (multiple choice)			44.107	0.000
A. White meat	79 (100.0%)	42 (42%)		
B. Red meat	21 (26.6%)	62 (68%)		
C. Seafood	10 (12.7%)	29 (29%)		
D. Animal offal	6 (7.6%)	20 (20%)		
Q14. How many eggs do you eat daily?			10.490	0.005
A. 0	20 (25.3%)	21 (21%)		
B. 1	35 (44.3%)	66 (66%)		
C. 2 and more than 2	24 (35.4%)	13 (13%)		
Q15.How many milk and milk products you intake daily?			19.329	0.000
A. 0 g	17 (21.5%)	49 (49%)		
B. 1−250 g	44 (55.7%)	45 (45%)		
C. ≥ 251 g	18 (22.8%)	6 (6%)		
Q16. How many beans and beans products you intake daily?			6.514	0.039
A. 0−40 g	41 (51.9%)	64 (64%)		
B. 41−80 g	30 (38.0%)	34 (34%)		
C. ≥ 81 g	8 (10.1%)	2 (2%)		
Q17. How many fruits do you intake daily?			1.860	0.395
A. 0−100 g	38 (48.1%)	40 (40%)		
B. 101−200 g	33 (41.8%)	52 (52%)		
C. ≥ 201 g	8 (10.1%)	8 (8%)		
Q18. What kind of fruits you usually eat?			16.525	0.000
A. Low fructose fruit	57 (72.2%)	52 (52%)		
B. Fructose rich fruit	15 (19.0%)	46 (46%)		
C. Cherry	7 (8.9%)	2 (2%)		
Q19. How many vegetables do you intake daily?			31.691	0.000
A. 0−250 g	44 (55.7%)	32 (32%)		
B. 251−500 g	20 (25.3%)	65 (65%)		
C. ≥ 501 g	15 (19.0%)	3 (3%)		
Q20. How many grease do you intake daily?			19.639	0.000
A. 0−10 g	26 (32.9%)	12 (12%)		
B. 11−15 g	31 (39.2%)	30 (30%)		
C. 16−20 g	17 (21.5%)	49 (49%)		
D. ≥ 21 g	5 (6.3%)	9 (9%)		

### Comparison of adiponectin and creatinine between healthy and gout groups

In gout patients, adiponectin was found to be lower as compared to healthy volunteers. These differences were found to be statistically significant (*P* < 0.01), which suggested that the patients might be associated with abnormal glucose metabolism and insulin resistance. In addition, the statistically significant differences were recorded in the levels of creatinine between the two groups, wherein higher creatinine was recorded in the gout group (*P* < 0.01). These results suggested that patients with gout exhibited abnormal renal function (Table 4).

**Table 4:** Comparison of adiponectin and creatinine between two groups

	Healthy group (Group A)	Gout group (Group B)	Value *P*
Adiponectin (mg/L)	8.33 ± 6.14	6.65 ± 7.02	*P* < 0.01
Creatinine (mmol/L)	67.36 ± 13.90	104.86 ± 40.71	*P* < 0.01

### NMR analysis of abnormal serum metabolism in healthy and gout groups

**Fig. 1:**
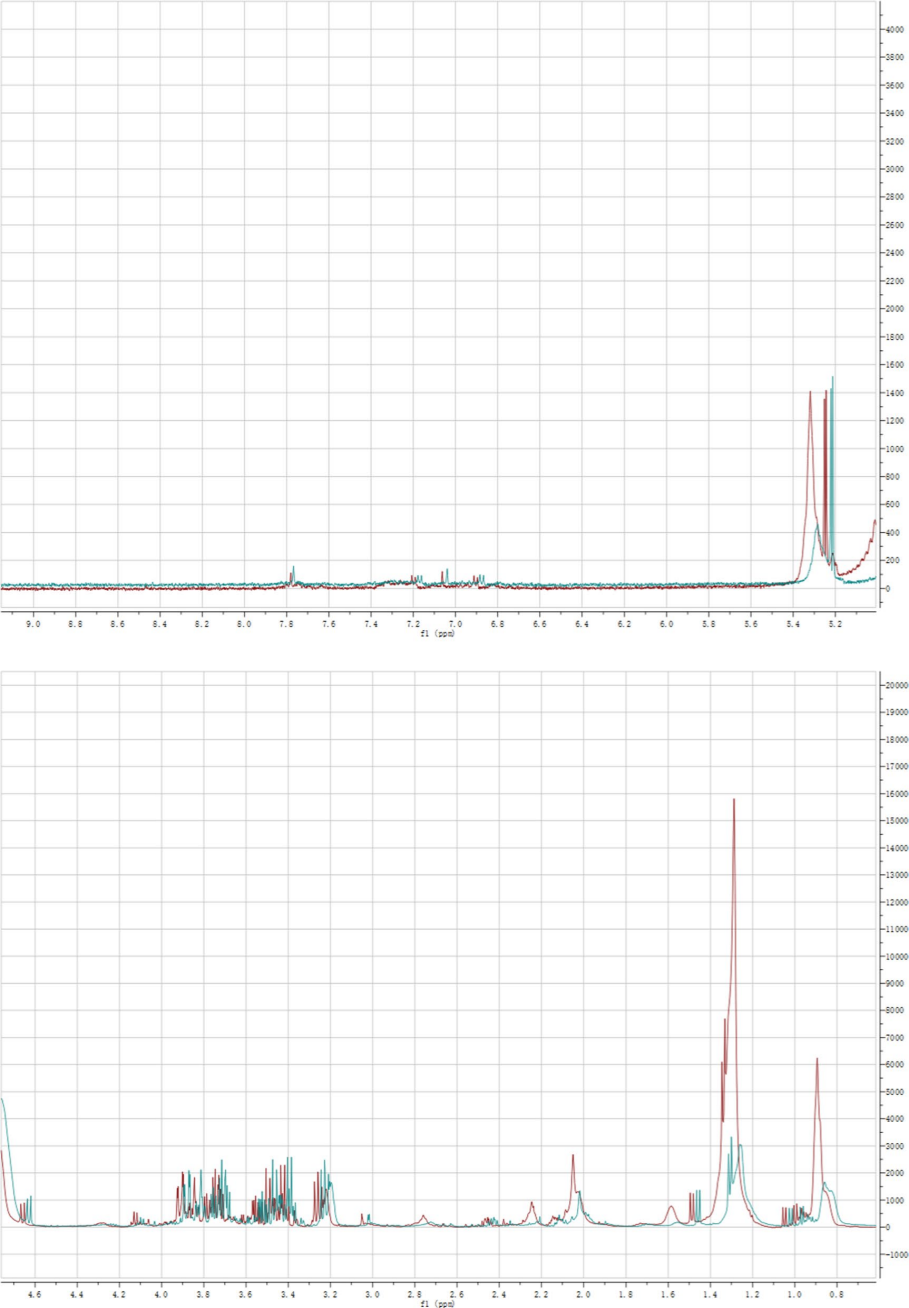
The NMR profiles of the serum samples from the gout group (red line) and the healthy group (green line)

The study analyzed the differences in ^1^H‐NMR spectra of serum metabolites between the healthy group and the gout group (Fig. 1). Referred relevant literature [8] and metabolite database HMDB (http://www.hmdb.ca), significant differences were identified in ^1^H‐NMR spectra. As shown in Fig. 1, 14 metabolites could be easily distinguished between the two groups. For metabolites with few differences, multivariate statistical analysis was required.

### Unsupervised PCA analysis of serum metabolites in healthy and gout groups

**Fig. 2:**
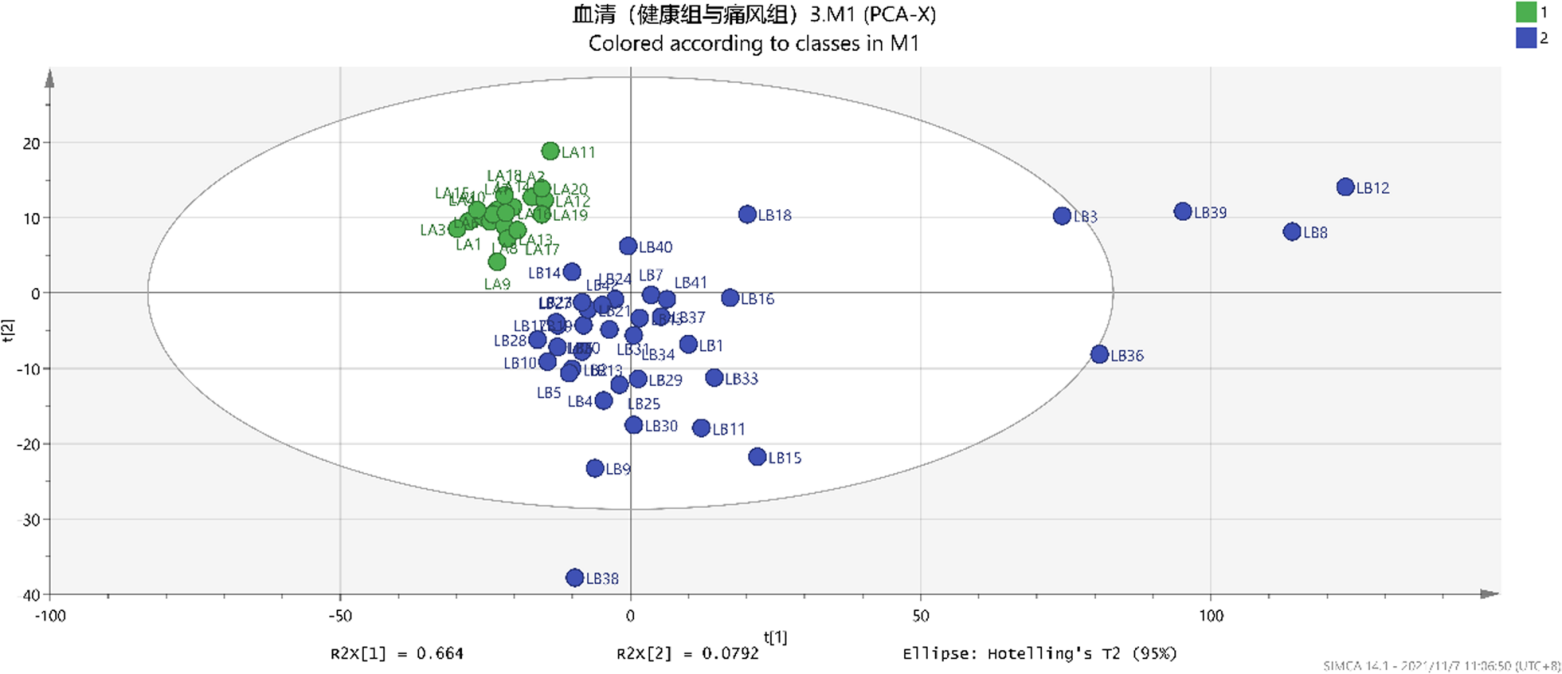
The gout group (  ) and healthy group (  ) in the PCA analysis diagram

The study conducted unsupervised PCA analysis for serum metabolites in healthy and gout groups to initial evaluation of prospective biomarkers. As shown in Fig. 2, healthy volunteers were found to exhibit a relatively stable metabolic state, which the distribution was relatively concentrated. The two groups were basically separated, however, some overlap was observed (PC1 vs. PC2, R2 = 90.5%, Q2 = 83.8%) (Fig. 2), which suggested the occurrence of a good pattern. The study further analyzed PLS‐DA and OPLS‐DA.

### PLS‐DA and OPLS‐DA analyses of serum metabolites in healthy and gout groups

**Fig. 3:**
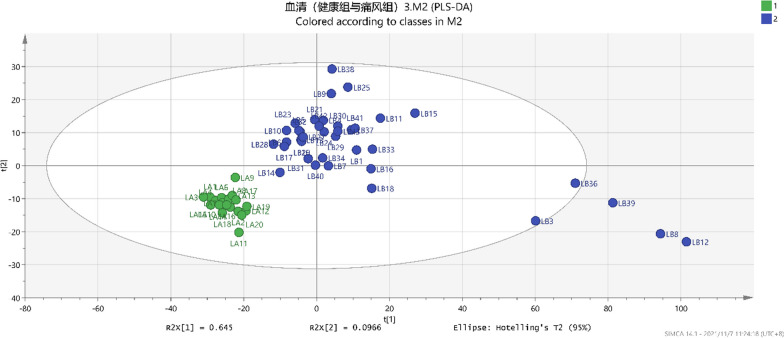
The gout group (  ) and healthy group (  ) in the PLS-DA analysis diagram

**Fig. 4:**
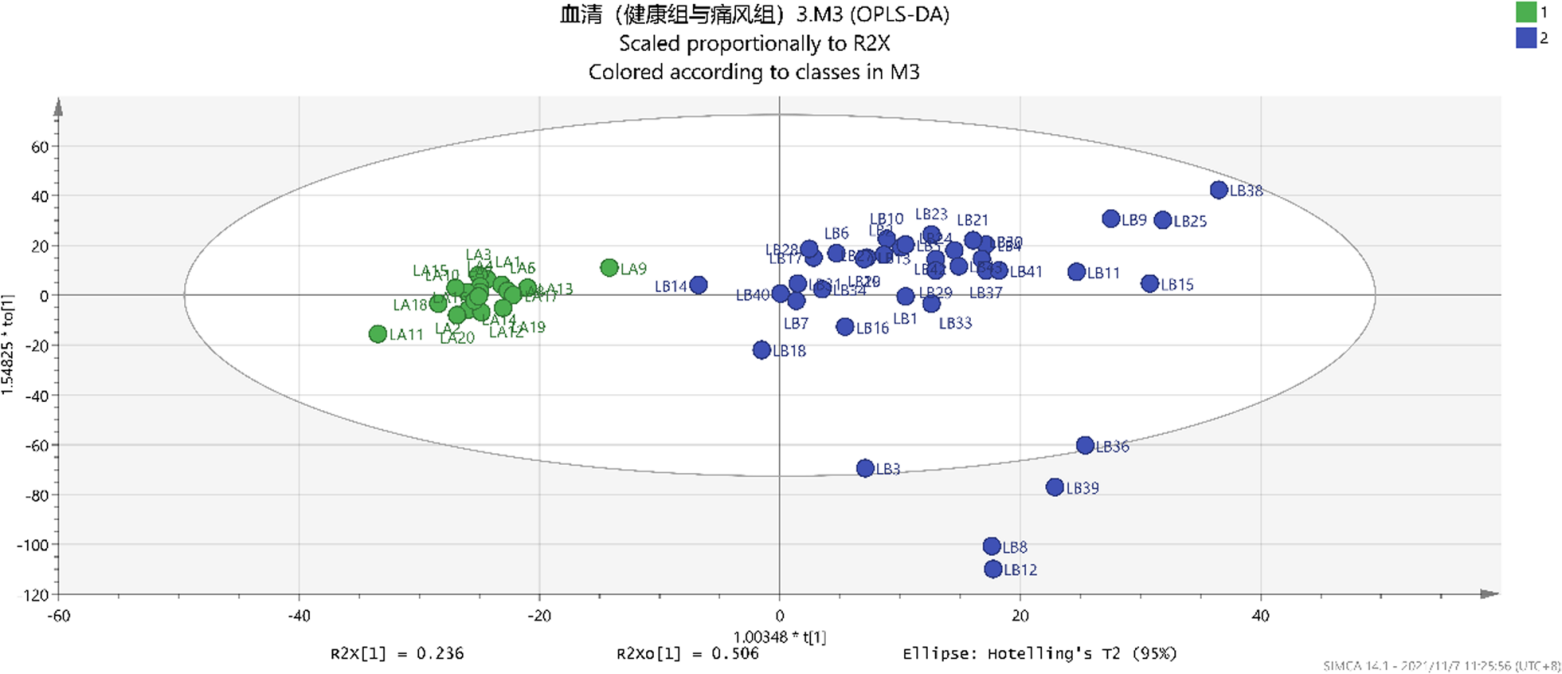
The gout group (  ) and healthy group (  ) in the OPLS-DA analysis diagram

The PLS-DA supervised pattern recognition method would be employed to further analyze the aforementioned data in order to enhance the optimization of the grouping model. The analysis results are shown in Fig. 3 [R2x = 74.2%, R2Y = 82.6%, Q2 (CUM) = 80.4%]. The score chart revealed a complete separation of the two groups. However, some samples were found to be relatively dispersed within the group. In order to mitigate the impact of confounding factors such as dietary habits, lifestyle choices, medication usage, individual variations, and other variables on the study groups while enhancing inter-group distinctions, we employed the widely utilized orthogonal signal correction (OSC) technique in conjunction with PLS-DA analysis to effectively reduce noise. Following this, OPLS‐DA analysis was performed, which eliminated system errors, filtered out irrelevant information, and enhanced the discriminative power of pattern recognition methods. The results are shown in Fig. 4 [R2x = 74.2%, R2Y = 82.6%, Q2 (CUM) = 80.9%]. Importantly, no crossover and overlap were observed between the groups, and the two groups were found to be distributed in a concentrated manner. These results showed that the model was effective. Incorporating established standards (VIP ≥ 1, *P* < 0.05), 47 metabolites were identified in combination with the actual map.

**Fig. 5:**
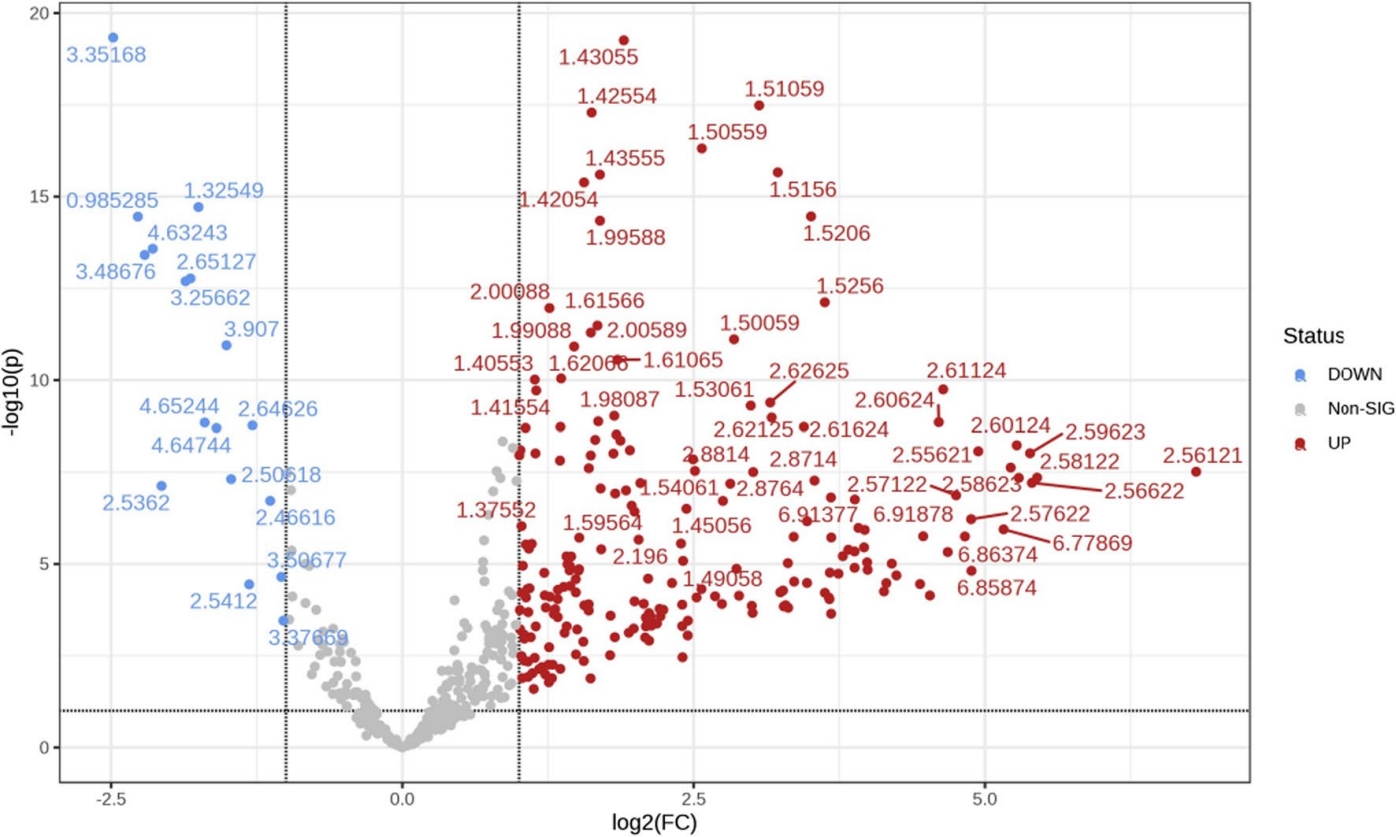
The Volcano Plot analysis between the gout group and healthy group

In the Volcano Plot analysis (Fig. 5, Table 5), compared with the gout group, the results shown that the levels of very low-density lipoprotein (VLDL)/low-density lipoprotein (LDL), lactic acid, prostaglandin D2, *N*‐acetyl glycoprotein, *O*‐acetyl glycoprotein, homocysteine, prostaglandin E2, 3‐hydroxy butyric acid, malic acid, succinic acid, creatinine phosphate, glycerol phosphate, betaine, trimethylamine, preserved ammonia acid, taurine, glycine, threonine, inositol, glycerol, glycerin, methionine, aspartic acid, creatinine, phenylalanine, methionine, histidine, isoleucine, alanine, leucine, lysine, acetic acid salt, and glutamate were found to be increased in the healthy group, while the levels of valine, proline, arginine and high-density lipoprotein (HDL) were decreased (Table 5).

**Table 5:** Names and changes in serum metabolites in the two study groups

Chemical shift value	Metabolite	VIP	Variation trend
0.82	HDL	1.1048	↓
0.98, 2.26	Valine	1.046	↓
3.25	Arginine	1.25	↓
3.35	Proline	1.01	↓
0.86	VLDL/LDL	1.073	↑
1.33	Lactic acid	0.53506	↑
1.38	Prostaglandin D2	1.29	↑
2.06	N-acetyl glycoprotein	1.16	↑
2.14	O-acetyl glycoprotein	1.17	↑
2.15	Homocysteine	1.27	↑
2.25	Prostaglandin E2	1.08	↑
2.31	3-Hydroxybutyric acid	1.35	↑
2.39	Malic acid	1.27	↑
2.4	Succinic acid	1.08	↑
3.12	Phosphocreatine	1.1	↑
3.22	Glycerol phosphocholine	1.16	↑
3.28, 3.26	Betaine	1.02	↑
3.42	Taurine	1.07	↑
3.57	Glycine	1.45	↑
3.58	Threonine	1.51	↑
3.63	Inositol	1.22	↑
3.64	Glycerine	1.29	↑
3.66	Glycerol	1.19	↑
3.87	Methionine	1.05	↑
3.89	Aspartic acid	1.21	↑
3.94	Creatine	1.41	↑
3.98	Phenylalanine	1.27	↑
0.93, 1.97, 3.65	Isoleucine	1.39	↑
1.48, 3.79	Alanine	1.488	↑
1.71, 0.94, 3.72	Leucine	1.34	↑
1.72, 3.01	Lysine	1.16	↑
1.91, 1.92	Acetate	1.21	↑
2.06, 2.35	Glutamate	1.31	↑
2.1, 2.41	Glutamine	1.06	↑
2.16, 2.17	Butyric acid	1.36	↑
2.22, 2.28	Acetoacetic acid	1.04	↑
2.24, 2.38	Acetone	1.28	↑
2.52, 2.55	Citric acid	1.05	↑
3.62, 4.65	D- glucose	1.36	↑
6.87, 7.22	Tyrosine	1.1	↑

### Analysis of potential metabolic pathways

**Fig. 6:**
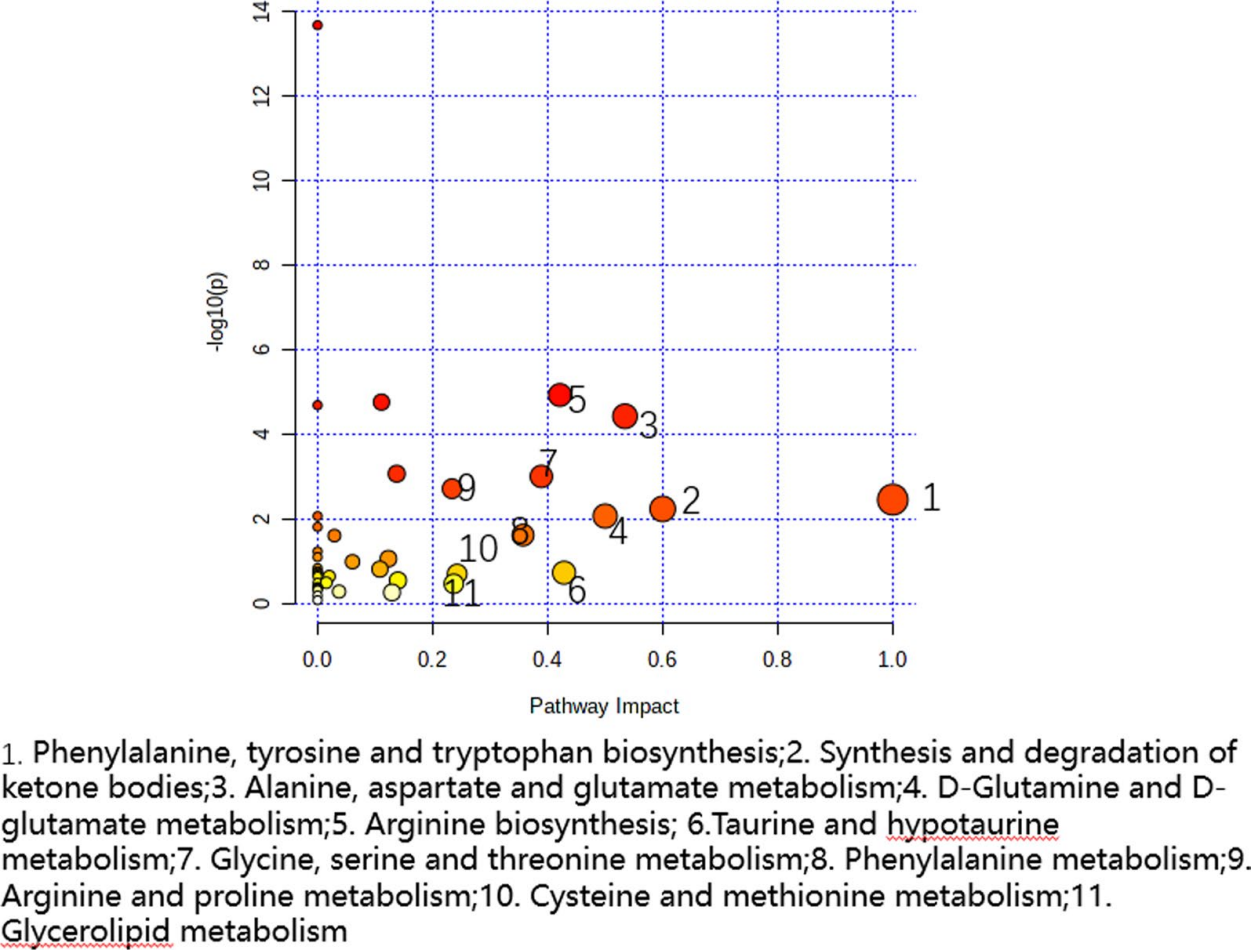
The metabolic network analysis diagram of the patients

The analysis of metabolite changes was further used to identify abnormal metabolic pathways. Briefly, 44 metabolic were putted into MetaboAnalyst 5.0, and the analysis identified 40 important metabolic pathways. Importantly, metabolic pathways with *P* value < 0.05 or Impact > 0.1 were identified as potential target pathways. Consequently, a total of 12 pathways were identified, which included ammonia acyl tRNA biosynthesis, biosynthesis of valine, leucine, and isoleucine, metabolism of alanine, glutamic acid, and aspartic acid, metabolism of glycine, serine, and threonine, phenylalanine, glycolysis/sugar dysplasia, synthesis and degradation of ketone body, D‐glutamine with D‐glutamine metabolism, citric acid cycle (TCA cycle), and triglyceride metabolism. When combined with the KEGG database, it was revealed that the metabolic pathways involved energy metabolism, glucose metabolism, amino acid metabolism, lipid metabolism, and immune disorders (Fig. 6, Table 6).

**Table 6:** Two groups of serum metabolites mainly involved in the metabolic pathway analysis results

	Hits	Raw *P*	LOG10(*P*)	Holm adjust	FDR	Impact
Ammonia acyl tRNA biosynthesis	15	5.15E−14	13.288	4.33E−12	4.33E−12	0
Valine, leucine, and isoleucine biosynthesis	4	2.48E−05	4.6061	0.002031	0.000694	0
Alanine, glutamic acid, and aspartic acid metabolism	6	4.98E−05	4.3026	0.004035	0.001046	0.53446
Glycine, serine, and threonine metabolism	5	0.001237	2.9078	0.096458	0.01484	0.38904
Phenylalanine, tyrosine, and tryptophan biosynthesis	2	0.003772	2.4234	0.29044	0.035239	1
Glycolysis/sugar dysplasia	4	0.003776	2.423	0.29044	0.035239	0.03011
Synthesis and degradation of ketone body	2	0.006184	2.2087	0.46381	0.051947	0.6
D-glutamine with D-glutamine metabolism	2	0.009125	2.0398	0.66613	0.058962	0.5
Metabolism of phenylalanine	2	0.025642	1.591	1	0.13462	0.35714
Citric acid cycle (TCA cycle)	2	0.092117	1.0357	1	0.40725	0.12311
Glutathione metabolism	2	0.16139	0.79212	1	0.61621	0.10839
Triglyceride metabolism	1	0.34321	0.46444	1	0.87362	0.23676

## Discussion

Refractory gout is a particular type of chronic gout, characterized by recurrent attacks and tophus formation, is related to the diet structures and exercise patterns of patients, and has specific biomarkers of biological metabolism. The questionnaire survey found that refractory gout female ratio was 20%, far higher than the literature in the general gout ratio of 5% [1], and they were all menopausal at the time of onset. It may be related to the loss of estrogen protection, uric acid excretion disorder, and weight gain after menopause, which makes gout attacks frequent and uric acid difficult to control. The present study conducted untargeted metabolomics identification studies on biomarkers related to refractory gout, identified 12 abnormal serum metabolic pathways, which are discussed below.

### Refractory gout requires adjustment of lifestyle

Adjusting lifestyle can help prevent gout attacks. Past research has indicated that a reasonable frequency of exercise can help prevent gout and promote uric acid metabolism.[9]. We also recorded that the exercise frequency was similar between the two groups. However, in terms of the selection of the exercise mode, the gout group tended to select confrontational movement, pedestrianism, hiking and so on, could lead to joints damage, promote uric acid deposition in damaged joints and induce gout attacks. Therefore, we advocate for patients with refractory gout to select exercises that have a lesser impact on joints, such as swimming and Tai chi. Other unhealthy lifestyle, such as smoking, retaining the sitting posture for long, and sleeping late, were noted to be greater in the gout group. It may be a risk factor for frequent gout attack.

### Refractory gout requires adjustment of the diet structures

A reasonable diet structures help prevent a gout attack. We noted that the gout group had a higher intake of grains and oils than the healthy group, although the literature suggests that increased intake of grains, especially high-fiber foods, can reduce uric acid crystallization [10], although there is no literature report supporting that recurrent refractory gout and formation of tophus are related to the intake of grains and oils. However, excessive intake of grains and oils can contribute to excessive total calorie intake, which easily creates an imbalance of carbohydrates, protein, and fat. In addition, we found that gout patients generally have lactate dehydrogenase (LDH) increased and adiponectin decreased, combined with abnormal lipid and glucose metabolism, which may affect uric acid metabolism. It is, therefore, necessary to control the total intake of both. Adjusting vegetable–fruit dietary pattern can delay gout attack [11]. We found that the gout group had lower vegetable intake than the healthy group, and the fruit intake was no statistically different relative to the healthy group. However, there were differences in fruit selection between the two groups. Gout patients tended to choose fruits with high sugar content, while high fructose intake was found to affect uric acid metabolism.

The gout group had significantly higher meat intake than the healthy group, who were more inclined to eat high-purine food such as red meat, seafood, and animal offal. The intake of milk, eggs, and beans in the gout group was lower than that in the healthy group, while the intake of potatoes was not statistically different between the groups. However, the above-mentioned diets do not increase uric acid metabolism according to the literature [12, 13]. These statistical differences may be attributed to the gout diets misunderstanding among gout patients, and it is a belief that milk, eggs, potatoes, and beans may increase purine metabolism and control intentionally. It is therefore suggested that we should strengthen the health education related to gout diet in future clinical work as well as advocate the reasonable distribution of food share on the basis of controlling the total calorie intake.

### Relationship between abnormal energy metabolism and acute attack of refractory gout

The present study revealed the involvement of abnormal energy metabolism in refractory gout. In addition, four pathways were found to be associated including citric acid cycle (TCA cycle), pyruvate metabolism, glycolysis/gluconeogenesis, and ketone body synthesis and degradation. Under normal physiological conditions, glucose performs aerobic metabolism in the mitochondria, which involves the breakdown of carbon dioxide and water. Pyruvate can be formed from glucose by glycolysis, and it can be converted back to glucose by gluconeogenesis (glycolysis/gluconeogenesis). In the presence of oxygen (aerobic respiration), pyruvate provides energy to the cells through the citric acid cycle (TCA cycle). When oxygen is limiting, pyruvate fermentation produces lactic acid and ketone bodies (the synthetic pathway of ketone bodies). The present study revealed increased levels of pyruvate in the serum of patients. At the same time, the levels of citric acid, α‐ketoglutaric acid, and acetoacetic acid were also found to be increased in the TCA cycle, which further resulted in the production of ketone bodies, such as hydroxybutyric acid and acetone. Importantly, the above four pathways are known to be located in the mitochondria. Therefore, it was speculated that patients with acute gout exhibited mitochondrial energy supply disorder, which blocked the glucose aerobic metabolism pathway in the patients. However, gout patients are often associated with abnormal glucose metabolism, even diabetes, and the two metabolic pathways affect each other. We found that patients tend to intake high sugar fruits and drinks and exercise less, which affects their purine metabolism.

The present study also reported elevated levels of alanine increased in the patients. Β‐alanine can transaminate with pyruvate to form malondialdehyde and L‐alanine. Malondialdehyde further gets converted into malonic acid, which is catalyzed by the action of malondialdehyde dehydrogenase. Following this, malonic acid gets converted into propylene acyl-coenzyme A and enters into the fatty acid biosynthesis. Since neuronal uptake and neuronal receptor sensitivity of β‐alanine have been previously demonstrated, β‐alanine might act as a pseudo‐transmitter in place of γ‐aminobutyric acid. Importantly, when alanine is present at high levels, it can act as a neurotoxin, mitochondrial toxin, and metabolic toxin. It has been previously reported that mitochondrial toxins induce the destruction of mitochondria and reduce cell respiration and oxidative phosphorylation of compounds [14]. Thus, an abnormal alanine metabolism pathway would affect mitochondrial functions. Alanine was also sourced from acetic acid. We found that the gout group drank more alcohol than the healthy group, especially associated with gout attacks induced by spirits and beer, which needs to enter TCA cycle to be fully metabolized into carbon dioxide and water. The study revealed that the level of serum acetic acid was increased in the gout group, which may be related to the disorder of TCA cycle, and the increase in acetic acid can promote the deposition of uric acid crystals in various parts of the human body [15].

### Relationship between lipid metabolism pathway and acute attack of refractory gout

The present study reported abnormal lipid metabolism is related to refractory gout recurrent attacks, which was related to the glycerophospholipid metabolism pathway and arachidonic acid metabolism pathway. Glycerophospholipid is one of the most abundant phospholipids present in the body. It is known to act as a biofilm. In fact, it is one of the ingredients of bile and membrane surface-active substances. Glycerol, HDL, LDL, and phosphocholine are known to be involved in the glycerol phospholipid metabolism pathway. It has been previously reported that hypercholesterolemia and hypertriglyceridemia synergistically affect reactive oxygen species (ROS) with uric acid, which further aggravates vascular endothelial cell injury and increases the risk of cardiovascular and cerebrovascular accidents. HDL, which carries a variety of enzymes, globulin, microRNA, complement components, and heterogeneous lipoprotein of reactants in the acute phase [16], is known to act as a protective predictor of high uric acid levels in gout [17]. The present study reported increased levels of glycerol, LDL, and phosphocholine in the gout group, while the levels of HDL were decreased, which indicated abnormal lipid metabolism in the patients.

It has been previously reported that abnormal lipid metabolism affects the sphingolipid metabolic pathway. Previous studies revealed that excessive cholesterol intake in hamsters significantly increased the levels of serine palmitoyl transferase and ceramide in bile, which further suggested that cholesterol regulated the sphingolipid metabolic pathway [18]. In a previous study, Shang et al. reported increased levels of sphingolipid and ceramides in patients with acute gout, suggesting that the onset of gout was related to the sphingolipid metabolism pathway [19]. Acute onset of gout is known to be related to gout crystals that stimulate the formation of NALP3 inflammasome by monocytes and promote the release of inflammatory factors, including Interleukin-2 (IL‐2) and tumor necrosis factor-α (TNF‐α), to act on joint synovium, which further causes inflammation expansion [20]. TNF‐α is an inflammatory factor that can stimulate the production of sphingolipid by the mitochondria. It has been previously reported that TNF‐α acts as a mediator of inflammatory responses and induces important cell signals in inflammatory pathways.

Glycerol phospholipid metabolism is known to disturb lipid metabolism in patients with gout, which involves arachidonic acid metabolism. Consequently, it promotes proliferation and induces inflammation. Lecithin cholesterol acyltransferase (LCAT) usually degrades phosphocholine (PC) to produce lysophosphatidylcholine (LPC). Lysophospholipase I (LYPLA1) can further degrade lysophosphatidylcholine into glycerophosphocholine (GPC) and produces arachidonic acid and other fatty acids. The present study explored increased serum PC and GPC levels in patients with acute gout. A large number of studies have previously shown that arachidonic acid and other fatty acids are increased in patients with acute gout [21]. Arachidonic acid is known to be involved in the synthesis of prostaglandin (PGE2). Importantly, this might be related to caspase‐1 mediated activation of cycde‐2 (COX‐2), which has been previously reported to be involved in the synthesis of PGE2 through the release of IL‐1β, by the protein precursor [22]. In the present study, PGE2 was found to be elevated in patients with gout, which suggested that it might be a potential target for inflammatory storms.

This study's results revealed that the intake of oils and beverages in the gout group was greater than that in the healthy group, which may be attributed to the fact that saturated fatty acids and triglycerides can cooperate with uric acid crystallization and induce monocytes to promote the release of Interleukin-1β (IL‐1β) and Interleukin-8 (IL‐8), which in turn further promotes the chemotaxis of neutrophils. The present study reported that the patients were associated with higher fatty acid metabolism, and these results were consistent with the findings of previous studies [23]. Altogether, these results suggested that abnormal fatty acid metabolism might act as a potential target for the disease. The present study also reported a negative correlation between serum adiponectin levels and serum uric acid, which might be related to the destruction of xanthine oxidase and other oxidants. It has been previously reported that adiponectin could improve insulin resistance, resist arteriosclerosis, and regulate lipid metabolism. In patients with gout, it might exert an anti‐inflammatory effect. In a previous study, Chengfei et al. reported that adiponectin affected downstream inflammatory signaling pathways in patients with gout, through the Adipo R2 signaling pathway, which ultimately reduced the production and secretion of IL‐1β in inflammatory cells. Thus, it exhibited a dose‐response relationship and played an anti‐inflammatory role in the inflammatory process of patients with gout [24].

### Relationship between amino acid metabolic pathways and acute attack of refractory gout

The present study identified four abnormal amino acid metabolic pathways in refractory gout, which included metabolism of glycine, serine, and threonine, glutathione metabolism, aminoacyl tRNA biosynthesis, and biosynthesis of valine, leucine, and isoleucine.

In general, glycine is synthesized from serine. Besides this, it can also be obtained from threonine, choline, or hydroxyproline [25]. At the same time, glycine can be decomposed into pyruvate and acetoacetic acid, which get translated into ɑ‐ketone glutaric acid. Both these compounds further participate in a tricarboxylic acid cycle and provide energy for the body. It has been previously reported that glycine exerted significant anti‐inflammatory effects, which involved inhibition of activation of nuclear transcription factor NF‐κB, degradation of IKB‐α, and production of Interleukin-6 (IL‐6) [26]. In the present study, increased levels of threonine, choline, serine, and glycine were reported in patients with gout, which suggested that abnormal metabolism of threonine, serine, and glycine might be associated with inflammatory stimulation, body dysfunction, and compensatory effects that enhanced the anti‐inflammatory response of glycine. The study further reported increased serum and fecal glutamate levels in patients with gout. Importantly, glutamic acid, as an organic acid, can lead to metabolic acidosis and tissue damage. It is even known to affect renal acid‐base balance metabolism, which further results in renal function damage [27]. In the present study, elevated creatinine levels were reported in patients with gout as compared to the healthy group, which indicated combined kidney damage. The raw materials involved in the synthesis of antioxidant glutathione are known to cause metabolic disorders of glycine and glutamate that affect the glutathione metabolic pathway. Importantly, it has been previously shown that this pathway can scavenge free radicals in the body and protect sulfhydryl groups in protein molecules [28].

The present study reported increased levels of phenylalanine, aspartic acid, methionine, isoleucine, leucine, and threonine in patients with refractory gout, which suggested abnormal metabolism of amino acids. This might be related to abnormal energy metabolism in patients with gout, which further leads to biosynthesis disorder of aminoacyl tRNA, and regulates inflammatory response [29].

Branched-chain amino acids (BCAAs) include isoleucine, leucine, and valine. The present study reported increased levels of isoleucine and leucine in the serum, while the levels of valine decreased, which indicated branch chain amino acid metabolism disorder in patients with an acute attack of gout. The metabolism of branched amino acids is known to be closely related to insulin resistance, lipid metabolism disorder, and anti‐inflammatory response. In a previous study, Eri et al. reported that BCAA increased postprandial plasma glucagon levels [30]. In another study, it was reported that patients with gout, whose isoleucine and leucine were elevated and associated with BMI and fasting glucose [31], exhibited blood glucose abnormalities. This study's results suggest that the BMI of the gout group was higher than that of the healthy group, implying that obesity is associated with gout attack and possibly the reduction of mitochondrial glucose utilization. In general, BCAAs can be transaminated to produce nitrogen, which is used for the synthesis of non‐essential amino acids, such as glutamine and alanine. The present study reported elevated levels of both these products. Alanine is generally used in protein synthesis. Additionally, it is used as a precursor for hepatic gluconeogenesis. During starvation, alanine is generated from BCAAs in muscle, and it is transported to the liver, where it is used for the glucose–alanine cycle, which manufactures glucose to fulfill the energy needs. Elevated levels of BCAAs can also lead to an increased inflammatory response. In fact, a high concentration of BCAAs is known to promote oxidative stress and a pro‐inflammatory state of peripheral blood mononuclear cells [32]. Recent studies also reported elevated levels of BCAAs in rheumatoid arthritis patients [33]. BCAAs can be transaminated into glutamine and be docked with modifier of snc (MOS), which formed five hydrogen bonds with amino acid residues THR1010, ARG880 and GLU802. Thus, it prevents substrate xanthine entries into the active site and plays a competitive role in inhibiting xanthine oxidase (XOD) into uric acid [34]. The present study reported a significant increase in glutamine, suggesting that BCAAs could induce abnormal purine metabolism.

## Conclusions

Altogether, there are some differences in the diet structures and lifestyle between refractory gout patients and healthy people, and the metabolites change tends to be involved in ammonia acyl tRNA biosynthesis, biosynthesis of valine, leucine, and isoleucine, metabolism of alanine, glutamic acid, and aspartic acid metabolism, metabolism of glycine, serine, and threonine, phenylalanine, glycolysis/sugar dysplasia, synthesis and degradation of ketone body, d‐glutamine with d‐glutamine metabolism, citric acid cycle (TCA cycle), triglyceride metabolism, and others. It suggests that patients with refractory gout have mitochondrial energy metabolism disorders, resulting in abnormal glucose metabolism, lipid metabolism and amino acid metabolism. Therefore, it is recommended to adjust diets and exercise patterns to protect joints, promote uric acid elimination and reduce acute gout attacks.

The scope of this study was limited to serum examination, and future improvements can be made in exploring more specific metabolic pathways by examining metabolites such as stool and urine.

## Data Availability

All data generated or analyzed during this study are included in this article. Further enquiries can be directed to the corresponding author.

## References

[1] Towiwat P, Chhana A, Dalbeth N (2019). The anatomical pathology of gout: a systematic literature review. BMC Musculoskel Disord.

[2] Schlee S, Bollheimer LC, Bertsch T, Sieber CC, Härle P (2017). Crystal arthritides–gout and calcium pyrophosphate arthritis. Zeitschrift für Gerontologie und Geriatrie..

[3] Zhu J, Zhao Y, Xu D. Frequently asked questions of gout(4): complication and concomitant disease. Zhonghua nei ke za zhi. 2018;57(12):930–1. 10.3760/cma.j.issn.0578-1426.2018.12.011 30486564

[4] Neogi T, Jansen TLTA, Dalbeth N, Fransen J, Schumacher HR, Berendsen D (2015). 2015 Gout classification criteria: an american college of Rheumatology/European league against Rheumatism collaborative initiative. Arthritis Rheumatol (Hoboken, NJ).

[5] Vatutin NT, Smyrnova GS, El-Khatib MA (2016). New gout classification criteria (ACR/EULAR 2015). ArhivʺVnutrennej Mediciny[J].

[6] Dehlin M, Jacobsson L, Roddy E (2020). Global epidemiology of gout: prevalence, incidence, treatment patterns and risk factors. Nat Rev Rheumatol.

[7] Bollard ME, Contel NR, Ebbels TMD, Smith L, Beckonert O, Cantor GH (2009). NMR-based metabolic profiling identifies biomarkers of liver regeneration following partial hepatectomy in the rat. J Proteome Res.

[8] Yang H, Robinson PN, Wang K (2015). Phenolyzer: phenotype-based prioritization of candidate genes for human diseases. Nat Methods.

[9] Song YL (2020). Effects of different exercise frequency on the incidence of gout, the serum levels of creatinine and uric acid as well as urinary PH value in patients with hyperuricemia. Hebei Med J.

[10] Major TJ, Topless RK, Dalbeth N, Merriman TR (2018). Evaluation of the diet wide contribution to serum urate levels: meta-analysis of population based cohorts. BMJ.

[11] Omar NSC, Abu Bakar FI, Abu Bakar MF, Yee LS, Mutalib MA. Antigout potential of selected Malaysian traditional vegetables/Ulam. Asian J Chem. 2021;33(11). 10.14233/AJCHEM.2021.2341.

[12] Rebholz CM, Crews DC, Grams ME, Steffen LM, Levey AS, Miller ER, Appel LJ, Coresh J (2016). DASH (Dietary Approaches to Stop Hypertension) diet and risk of subsequent kidney disease. Am J kidney Dis.

[13] American Diabetes Association (2019). Lifestyle management: standards of medical care in diabetes-2019. Diabetes Care.

[14] Shetewy A, Shimada-Takaura K, Warner D, Jong CJ, Mehdi A-BA, Alexeyev M (2016). Mitochondrial defects associated with β-alanine toxicity: relevance to hyper-beta-alaninemia. Mol Cell Biochem.

[15] Liu YH, Cheng R, Ou CY, Zhang XD, Fu TM (2020). Acetate: an alcohol metabolite as a growth promoter of pathological crystallization of gout. Cryst Growth Des.

[16] Toth PP, Barter PJ, Rosenson RS, Boden WE, Chapman MJ, Cuchel M (2013). High-density lipoproteins: a consensus statement from the National Lipid Association. J Clin Lipidol.

[17] Liang J, Jiang Y, Huang Y, Song W, Li X, Huang Y (2020). The comparison of dyslipidemia and serum uric acid in patients with gout and asymptomatic hyperuricemia: a cross-sectional study. Lipids Health Dis.

[18] Shin H-W, Kim D, Lee Y, Yoo H-S, Lee BJ, Kim JS (2009). Alteration of sphingolipid metabolism and pSTAT3 expression by dietary cholesterol in the gallbladder of hamsters. Arch Pharmacal Res.

[19] Lyu S, Ding R, Liu P, OuYang H, Feng Y, Rao Y (2019). LC-MS analysis of serum for the metabolomic investigation of the effects of pulchinenoside b4 administration in monosodium urate crystal-induced gouty arthritis rat model. Molecules.

[20] Zhong S, Wang ZY, Guo LW, Lin Y (2021). The value of NALP3 inflammasome expression and related indexes in patients with actue gout. Int J Lab Med.

[21] Deng J (2020). The mechanism on Chinese medicine Huzhen Tongfeng formula on acute gouty arthritis. Guangdong Pharm Univ.

[22] Kim Y, Gromovsky AD, Brown JM, Chung S (2018). Gamma-tocotrienol attenuates the aberrant lipid mediator production in NLRP3 inflammasome-stimulated macrophages. J Nutr Biochem.

[23] Wang Y, Bi C, Pang W, Liu Y, Yuan Y, Zhao H (2019). Plasma metabolic profiling analysis of gout party on acute gout arthritis rats based on UHPLC-Q-TOF/MS combined with multivariate statistical analysis. Int J Mol Sci.

[24] Su J, Chai KJ, Li ZQ (2015). Expression of adiponectin and peroxisome proliferator activated receptor γ in patients with primary gouty arthritis and asymptomatic hyperuricemia. Qinghai Med J.

[25] McCarty MF, O'Keefe JH, DiNicolantonio JJ (2018). Dietary glycine is rate-limiting for glutathione synthesis and may have broad potential for health protection. Ochsner J.

[26] Quirke A-M, Fisher BAC, Kinloch AJ, Venables PJ (2011). Citrullination of autoantigens: upstream of TNFα in the pathogenesis of rheumatoid arthritis. FEBS Lett.

[27] Liss DB, Paden MS, Schwarz ES, Mullins ME (2013). What is the clinical significance of 5-oxoproline (pyroglutamic acid) in high anion gap metabolic acidosis following paracetamol (acetaminophen) exposure?. Clin Toxicol.

[28] Alia G, Bella A, Daniel P, Alexandra CF, Ifat A, Fernandez GJ, James T, Shani D, Isabelle FS, Natan W, Eugene CY, Oren R, Inbal M, Eyal G. Fatty liver-mediated glycine restriction impairs glutathione synthesis and causes hypersensitization to acetaminophen. bioRxiv: the preprint server for biology. 2023. 10.1101/2023.01.16.524043.

[29] Fagan SG, Helm M, Prehn JHM (2021). tRNA-derived fragments: a new class of non-coding RNA with key roles in nervous system function and dysfunction. Prog Neurobiol.

[30] Wada E, Kobayashi M, Kohno D, Kikuchi O, Suga T, Matsui S (2021). Disordered branched chain amino acid catabolism in pancreatic islets is associated with postprandial hypersecretion of glucagon in diabetic mice. J Nutr Biochem.

[31] Zhang Y, Zhang H, Chang D, Guo F, Pan H, Yang Y (2018). Metabolomics approach by (1)H NMR spectroscopy of serum reveals progression axes for asymptomatic hyperuricemia and gout. Arthritis Res Ther.

[32] Zhenyukh O, Civantos E, Ruiz-Ortega M, Sánchez MS, Vázquez C, Peiró C (2017). High concentration of branched-chain amino acids promotes oxidative stress, inflammation and migration of human peripheral blood mononuclear cells via mTORC1 activation. Free Radic Biol Med.

[33] Narasimhan R, Coras R, Rosenthal SB, Sweeney SR, Lodi A, Tiziani S (2018). Serum metabolomic profiling predicts synovial gene expression in rheumatoid arthritis. Arthritis Res Ther.

[34] Chen Y (2022). Alleviating effect of green tea extract with high amino acid on hyperuricemia through the hepato-kidney-intestinal axis and molecular docking study. Southwest Univ.

